# Cesarean section delivery is a risk factor of autism-related behaviors in mice

**DOI:** 10.1038/s41598-021-88437-8

**Published:** 2021-04-26

**Authors:** Masatoshi Nagano, Fumihito Saitow, Shinpei Higo, Makoto Uzuki, Yasunori Mikahara, Toshio Akimoto, Hitoshi Ozawa, Katsuhiko Nishimori, Hidenori Suzuki

**Affiliations:** 1 Department of Pharmacology, Graduate School of Medicine, Nippon Medical School, 1-1-5 Sendagi, Bunkyo-ku, Tokyo, Japan; 2 Department of Anatomy and Neurobiology, Graduate School of Medicine, Nippon Medical School, Tokyo, Japan; 3 Division of Laboratory Animal Science, Nippon Medical School, Tokyo, Japan; 4 Department of Obesity and Internal Inflammation, Fukushima Medical University, Hikarigaoka 1, Fukushima, Fukushima Japan

**Keywords:** Neuroscience, Psychology, Diseases, Pathogenesis

## Abstract

Cesarean section (C/S) is one way of delivering babies, and is chosen when mothers or babies are facing problems or life-threatening conditions during pregnancy. Many meta-analyses have suggested an etiological relationship between C/S delivery and autism spectrum disorders (ASDs). However, as a risk factor for ASDs, C/S delivery has not yet been well studied. Because C/S deliveries have been increasing, it is very important to investigate the causal association between C/S and ASDs. Here, using three approaches, we showed experimentally that C/S delivery induced ASD-like traits in offspring mice, and that some of these changes were ameliorated by one-time oxytocin (OXT) treatment. Treatment with OXT receptor antagonists before natural delivery also induced ASD-related behaviors. Moreover, wild-type mice born to OXT-KO dams showed similar changes. Thus, insufficient OXT exposure from dams to offspring during delivery may be a trigger for ASD-related behaviors.

## Introduction

As with any surgery, cesarean sections (C/Ss) are associated with some risks for the health of both mothers and children, as well as for that of future pregnancies. The World Health Organization recommends non-clinical interventions to reduce unnecessary C/Ss^1^. However, with the progress of reproductive medicine, cases that require C/S delivery are increasing, such as late births or births after fertility treatments. An etiological relationship between C/S delivery and autism spectrum disorders (ASDs) has been suggested^2,3^. Both the environment and many genes have been recognized as causal factors of ASDs^4^. Of these, the oxytocin (OXT) receptor gene is associated with ASDs^4–6^; OXT is a key molecule that acts as a uterine-contracting hormone during delivery. Furthermore, OXT is known as a regulator of social behavior^7^ and is a positive candidate therapeutic tool for ASD^8^. However, the possibility of C/S delivery as a risk factor for ASDs has not yet been well studied. Because C/S delivery rates are increasing, it is crucial to investigate any causal association between C/S and ASDs. Moreover, if there is an association, the ASD-inducing mechanism of C/S will urgently need to be revealed so that the incidence of ASDs can be reduced. Thus, we experimentally examined the effects of C/S on the development of offspring, and especially on the risk of ASD-related behavior, in mice. We also investigated the underlying mechanisms and the potential to ameliorate these effects. We previously demonstrated that postnatal OXT treatment is effective for ameliorating sociability in an ASD model mouse, *15q dup*^9^. Here, we again examined the effects of OXT treatment on the ASD-related behaviors induced in the offspring. Through this work, we revealed the importance of OXT exposure from dams to pups during delivery for the suppression of ASD-related behaviors in offspring.

## Results

### Effects of C/S delivery on social behaviors in both male and female offspring

**Figure 1:**
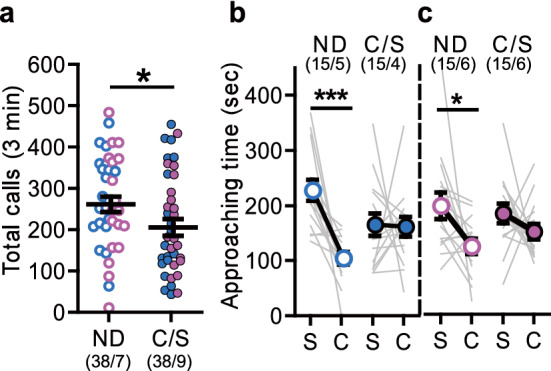
Effects of cesarean section (C/S) delivery on pups’ sociability. (**a**) Number of ultra-sonic vocalization (USV) calls at postnatal day (PD) 8 (naturally delivered [ND]: male = 19, female = 19, C/S delivered [C/S]: male = 19, female = 19; *p* = 0.039). (**b**,**c**) Approaching time to the stranger cage (S) and the empty cage (C) in the three-chamber social interaction (3-CSI) test. [(**b**) male, ND: *p* < 0.001, C/S: *p* = 0.751; (**c**) female, ND: *p* = 0.030, C/S: *p* = 0.303]. Male offspring, blue circles; female offspring, pink circles. Data represent the mean ± SEM. The numbers in parentheses indicate the numbers of mice tested and their litters (mice/litters) in each group. *p < 0.05, ***p < 0.001.

We first examined the effects of C/S on offspring sociability compared with naturally delivered (ND) mice. The numbers of ultra-sonic vocalization (USV) calls emitted by pups separated from their foster dams, ICR mice, were measured at postnatal day (PD) 8. The total number of calls of the C/S mice was significantly lower than that of ND mice (Fig. 1a). At 8 weeks of age, the ND mice spent significantly more time around stranger mice than around the empty cage in the three-chamber social interaction (3-CSI) test, whereas the C/S mice did not; this was true in both male and female mice (Fig. 1b,c). Thus, the mice born by C/S delivery showed social abnormalities.

### Effects of either OXT receptor antagonist treatment just before natural delivery or one-time OXT treatment on ASD-related behaviors in offspring

**Figure 2:**
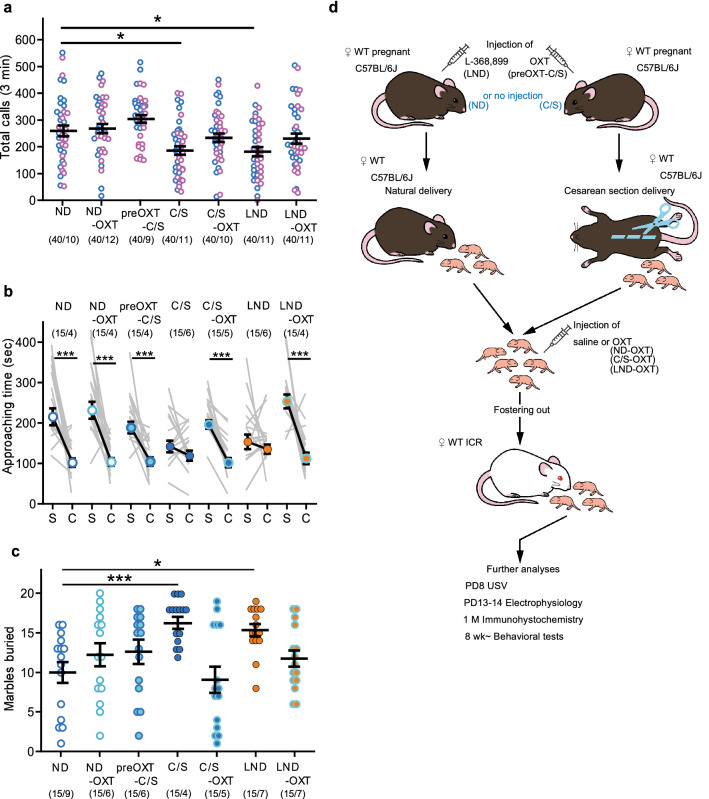
Effects of perinatal oxytocin (OXT) or prenatal OXT receptor antagonist treatment on offspring behaviors. (**a**) Number of USV calls at PD 8 (in all seven groups, *n* = 40: male = 20, female = 20; ND vs. C/S, *p* = 0.016; ND vs. born to L-368,899-treated dams via natural delivery [LND], *p* = 0.009). (**b**) Approaching time of male mice to the stranger cage (S) and the empty cage (C) in the 3-CSI test (ND: *p* = 0.001, ND-OXT: *p* < 0.001, preOXT-C/S: *p* < 0.001, *p* = 0.268, C/S-OXT: *p* < 0.001, LND: *p* = 0.804, LND-OXT: *p* < 0.001). (**c**) Number of marbles buried by male mice in the marble burying (MB) test (ND vs. C/S: *p* < 0.001, ND vs. LND: *p* = 0.035). Data represent the mean ± SEM. The numbers in parentheses indicate the numbers of mice tested and their litters (mice/litters) in each group. *p < 0.05, ***p < 0.001.

During pregnancy, plasma OXT levels in dams are only elevated at the expulsive phase on the day of delivery in both rats^10^ and mice^11^. These studies suggest that C/S delivery results in a large decrease in OXT exposure for rodent fetuses in the uterus at birth. In addition, it has been reported that OXT during the delivery process is important for mediating GABAergic inhibition, which suppresses the emergence of autistic features in offspring^12^. Thus, insufficient OXT exposure during delivery may be the cause of the social abnormalities that were observed in C/S-delivered offspring (described in the previous section). To confirm this hypothesis, we applied pharmacological challenges (Fig. 2d).

We first examined the effects of OXT receptor agonists and antagonists on the behaviors of C/S-delivered mice. For USV calls, just one postnatal or prenatal OXT treatment was able to suppress the C/S-induced decrease (Fig. 2a). In addition, we examined the effects of the OXT-receptor antagonist L-368,899 on the behaviors of ND mice. Before natural delivery, L-368,899 was injected into pregnant C57BL/6J mice. Mice born to the L-368,899-treated dams with natural delivery (LND) showed a similar reduction in USV calls as that of C/S-delivered mice, and postnatal OXT treatment recovered this reduction (Fig. 2a).

In the 3-CSI test, both prenatal and postnatal OXT treatments were able to recover the low sociability of C/S-delivered mice, both in male and female animals (Fig. 2b and Supplementary Fig. 2d). LND mice exhibited low sociability in the 3-CSI test, similar to that of C/S-delivered mice; this effect was recovered by postnatal OXT treatment (LND-OXT) in both sexes.

C/S-delivered male mice also exhibited a repetitive and persistent trait, which is one of the typical features of individuals with ASDs, in the marble burying (MB) test^13,14^. The number of marbles buried by C/S-delivered male mice was significantly higher than that of ND mice (Fig. 2c). This effect was not observed in female mice (Supplementary Fig. 2e). Thus, C/S delivery does not affect male and female mice equally. LND male mice also buried more marbles than ND mice. In the MB test, perinatal OXT treatment also effectively suppressed the increase in buried marbles that was observed in both C/S-delivered and LND mice.

The results of these pharmacological approaches support our hypothesis and suggest that the physical process of C/S delivery may not in itself be the cause of ASD-like traits.

In the other examined behavioral tests, such as the open-field (OF), light/dark (L/D), and fear-conditioning (FC) tests, a significant difference between ND and C/S mice was only observed in the FC test (Supplementary Figs. 1, 2). In female mice only, a significant increase in freezing was observed in C/S mice compared with ND mice (Supplementary Figs. 1g,h, 2g,h). Furthermore, in some ASD model mice, hypersensitivity to sound has been reported^15,16^. We therefore also examined sound hypersensitivity in male mice by measuring the startle response to loud sounds. However, we did not detect any hypersensitivity to 110 or 120 dB sounds in C/S mice (Supplementary Fig. 1d,e).

Postnatal OXT treatment did not markedly affect the ND mice (ND-OXT) in any of the examined behavioral tests (Supplementary Figs. 1, 2).

Other than behavioral changes, we also examined the effects of C/S delivery on the brains of offspring. In some ASD model rodents, elevated intracellular chloride concentrations ([Cl^–^]_i_) have been detected in neonatal hippocampal neurons, which is associated with the pathogenesis of ASDs^12^. Furthermore, maternal OXT during delivery suppresses the emergence of ASD-like features by reducing [Cl^–^]_i_^12^. However, we did not detect any differences in the Cl^–^ reversal potential in hippocampal neurons between ND and C/S mice at around PD15 (Supplementary Fig. 1i). Moreover, insufficient OXT seems to be critical during delivery only; at 1 month, we were unable to detect any differences in the numbers of OXT-immunoreactive cells in either the hypothalamic paraventricular nuclei (PVN) or the supraoptic nuclei (SON) between male ND and C/S mice (Supplementary Fig. 1j,k). This finding is different from that reported in another ASD mouse model, which was deficient in *Cntnap2*^17^.

### Behavioral effects in wild-type offspring born to OXT-knockout dams

**Figure 3:**
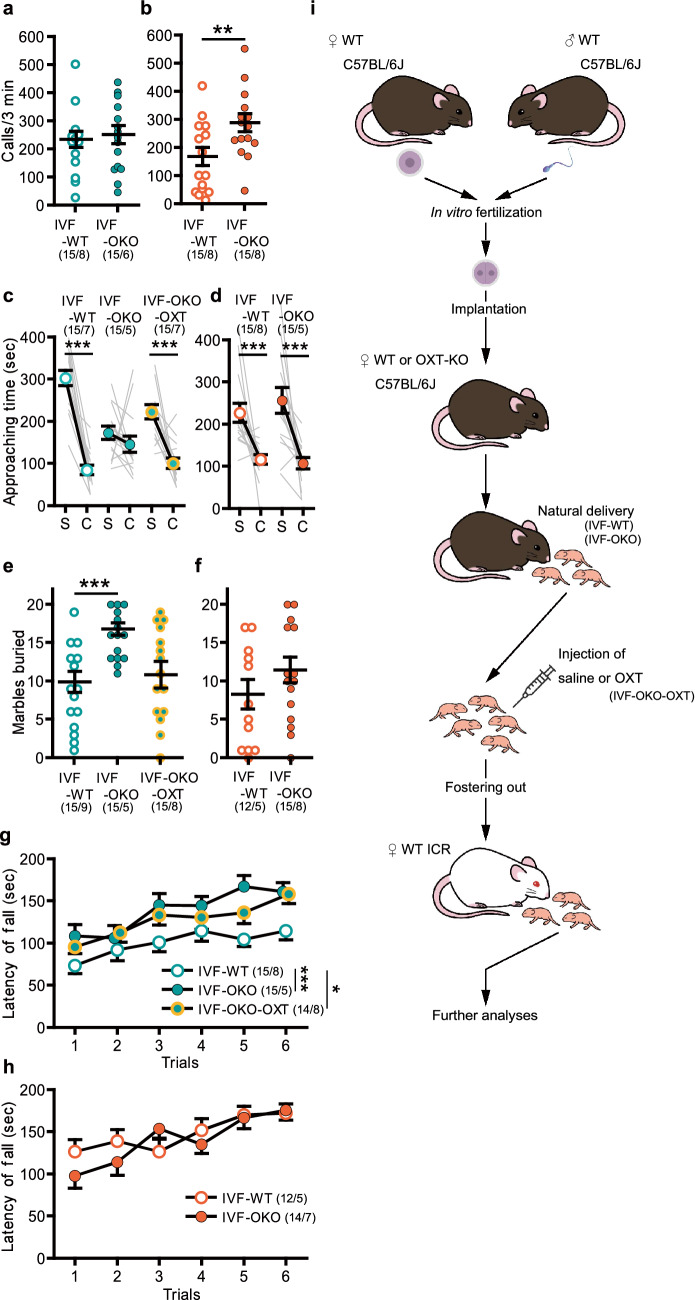
Effects of a lack of maternal OXT on the offspring. (**a**,**b**) Number of USV calls at PD 8. [(**a**) male, in vitro fertilization [IVF]-wild-type [WT] vs. IVF-OXT-knockout [OKO]: *p* = 0.681, (**b**) female, IVF-WT vs. IVF-OKO: *p* = 0.006]. (**c**,**d**) Approaching time to the stranger cage (S) and the empty cage (C) in the 3-CSI test. [(**c**) male, IVF-WT: *p* < 0.001, IVF-OKO: *p* = 0.124; IVF-OKO-OXT: *p* < 0.001, (**d**) female, IVF-WT: *p* < 0.001; IVF-OKO: *p* < 0.001]. (**e**,**f**) Number of marbles buried in the MB test. [(**e**) male, IVF-WT vs. IVF-OKO: *p* = 0.001, IVF-WT vs. IVF-OKO-OXT: *p* = 0.492, (**f**) female, IVF-WT vs. IVF-OKO: *p* = 0.223]. (**g**,**h**) Latency to fall from the rod in the rotarod test. [(**g**) male, body weight, IVF-WT: 26.7 ± 0.33 g, IVF-OKO: 28.1 ± 0.36 g, IVF-OKO-OXT: 26.7 ± 0.32 g; IVF-WT vs. IVF-OKO: *p* = 0.003, IVF-WT vs. IVF-OKO-OXT: *p* = 0.044, (**h**) female, two-way analysis of variance, group: *F*_1, 24_ = 0.399, *p* = 0.534]. (**i**) Schema of the procedures in this section. Data represent the mean ± standard error of the mean. The numbers in parentheses indicate the numbers of mice tested and their litters (mice/litters) in each group. *p < 0.05, **p < 0.01, ***p < 0.001.

Next, we re-examined our hypothesis using OXT-knockout (KO) mice. In vitro fertilized (IVF) wild-type (WT) C57BL/6J mouse embryos were implanted into pseudopregnant OXT-KO or WT female C57BL/6J mice. Because OXT-KO female mice show no deficits in gestation or parturition^18^, we were able to examine mice who did not receive OXT from their dams during delivery (Fig. 3i). Unexpectedly, the USV results of mice born to OXT-KO dams (IVF-OKO) were different from those of C/S mice. We did not detect any decrease in the total USV calls of IVF-OKO compared with mice born to WT dams (IVF-WT). Moreover, USV call numbers appeared to be different between the male and female mice. We therefore analyzed the data separately from one another. In males, there were no differences between IVF-WT and IVF-OKO mice (Fig. 3a). Surprisingly, in females, the USV call numbers of IVF-OKO mice were significantly higher than those of IVF-WT mice (Fig. 3b). In the 3-CSI test, IVF-WT mice showed normal sociability, and only male IVF-OKO mice (Fig. 3c), but not female IVF-OKO mice (Fig. 3d), showed lower sociability. Postnatal OXT treatment in IVF-OKO mice (IVF-OKO-OXT) attenuated the decrease in sociability of male IVF-OKO mice (Fig. 3c). In the MB test, the numbers of buried marbles were significantly higher in male IVF-OKO mice than in male IVF-WT mice (Fig. 3e). Postnatal OXT treatment of IVF-OKO mice (IVF-OKO-OXT) significantly reduced the numbers of marbles buried. Female IVF-OKO mice did not bury more marbles than IVF-WT mice in the MB tests (Fig. 3f). Furthermore, a significantly enhanced performance in the rotarod test, which has been previously observed in some ASD mouse models^19,20^, was observed in the male IVF-OKO mice (Fig. 3g) but not in the female IVF-OKO mice (Fig. 3h). Postnatal OXT treatment did not attenuate this change in the male IVF-OKO mice. In the FC test, an increased freezing ratio was observed in male IVF-OKO mice, but not in female IVF-OKO mice (Supplementary Figs. 3f,g, 4d,e). The increased freezing in male IVF-OKO mice was suppressed by postnatal OXT treatment.

## Discussion

Taken together, C/S delivery induced ASD-related behaviors in both male and female mice. Our results indicate that OXT exposure from the dam during delivery is critical for male mice. In contrast, in female mice, OXT exposure seems to be important, but not essential. The abnormalities observed in female C/S-delivered mice, which were attenuated by prenatal and postnatal OXT, may be attributed to a combination of factors that include OXT because female mice born to OXT-KO dams did not show ASD-related behaviors.

Sex differences were observed not only in the C/S-delivered mice, but also in the IVF-OKO mice. In the C/S mice, increased numbers of buried marbles in the MB test were observed only in male mice, and increased freezing in the FC test was observed only in female mice. In contrast, in the IVF-OKO mice, increased numbers of buried marbles in the MB test, better performance in the rotarod test, and increased freezing in the FC test were observed in male mice only, while USV calls increased in female mice only. Regarding OXT, this hormone has more roles in females, such as in uterine contraction and milk ejection. Furthermore, quantitative differences between the sexes have been observed in the mRNA expression levels of OXT and OXT receptors. In mice, these mRNA levels are higher in the female brain than in the male brain during the perinatal period^21^. This finding may indicate that the roles of OXT in the brain during the perinatal period are different between the sexes.

There were also some differences between C/S and IVF-OKO mice. One possible reason for such differences may be the quantity of OXT that fetuses receive from dams. In the uterus of OXT-KO mice, fetuses do not receive any OXT from their dams during pregnancy. However, in the case of C/S delivery, although the amount of OXT from the dams may be largely decreased compared with that of natural delivery, the fetuses are able to receive OXT during the period before delivery in the uterus^10^. Another possibility may be the result of a specific feature of OXT-KO mice. OXT-KO mice are hyper-reactive to stressors, and plasma corticosterone levels are enhanced when they are exposed to stressors^22,23^. Thus, OKT-KO dams might react to stressors easily during pregnancy and delivery, and this may affect the fetuses in utero. Prenatal trauma increases both the time and frequency of USV calls in pups^24^. Therefore, this may have been the reason for our inability to detect any decrease in USVs in the IVF-OKO mice. Regarding USV calls, the duration of pregnancy might also affect this trait because the day of measurement is close to the day of birth. Pregnancy duration is approximately 1 day longer in IVF cases than in cases of natural delivery.

In the present study, perinatal OXT treatment did not seem to induce adverse effects on offspring, especially in ND mice. However, some reports have mentioned a risk of perinatal OXT treatment in human children^25,26^. In clinical states, it may thus be important to pay more attention to perinatal OXT treatment.

In conclusion, insufficient maternal OXT exposure in fetuses induces ASD-related behaviors in mice, especially in male animals. In this sense, C/S delivery is a risk factor of ASD-related behaviors in the offspring because it largely diminishes the prenatal exposure of fetuses to OXT. These changes were reversible by perinatal OXT treatment. Female offspring seemed to be somewhat more resistant to the reduce of maternal OXT than male offspring. Exploring the mechanisms underlying this female resistance may contribute to our understanding of the lower incidence of ASDs in human females, and may also indicate new therapeutic strategies for ASDs.

## Materials and methods

### Mouse models and the implantation of in vitro fertilized eggs

WT C57BL/6J and ICR mice were purchased from Japan SLC (Shizuoka, Japan). In this study, ND mice from WT dams were designated ND mice. C/S was performed on gestational day (GD) 19 (full term). To avoid the effects of anesthesia, euthanasia was performed via cervical dislocation just before C/S. The mice delivered by C/S were designated C/S mice. OXT was treated perinatally, as described in the Drug treatment subsection. Prenatally OXT-treated C/S offspring were designated preOXT-C/S. OXT was also administered postnatally in ND and C/S mice, which were designated ND-OXT and C/S-OXT, respectively. L-368,899, an OXT receptor antagonist, was also administered prenatally. After treatment, ND pups were designated LND. Postnatally OXT-treated LND mice were designated LND-OXT mice.

OXT-KO C57BL/6J mice were obtained using sperm from OXT-KO mice supplied by Professor Nishimori of Fukushima Medical University^18^. We tested C57BL/6J mice born to WT or OXT-KO dams.

OXT-KO mice were used to examine the effects of OXT during delivery on the offspring. IVF of WT C57BL/6J mice was performed using eggs derived from 4- or 8-week-old superovulated females (*n* = 93) and sperm derived from 8-week-old males (*n* = 21). These IVF embryos were implanted to pseudopregnant WT or OXT-KO female C57BL/6J mice. The gestational duration of IVF mice was 20 days, 1 day longer than that of normal pregnancy. Naturally delivered mice from WT or OXT-KO dams at GD20 were used for comparison (pups were designated IVF-WT and IVF-OKO, respectively). Postnatally OXT-treated IVF-OKO mice were designated IVF-OKO-OXT mice.

All mice, including C/S, ND, IVF-WT, and IVF-OKO mice, were fostered from birth to weaning by ICR female mice who had just given birth (Figs. 2d, 3i) because the successful ratio of fostering in ICR mice is higher than in C57BL/6 fosters^27^. After weaning at PD21, the mice were divided into groups of two to five mice per cage. All mice were kept at a constant temperature (22 °C ± 1 °C) and a regular light/dark cycle (lights on from 06:00 to 20:00), with free access to food and water. All experiments were conducted in accordance with the National Institutes of Health Guide for the Care and Use of Laboratory Animals and ARRIVE guidelines. This study was reviewed by the Institutional Animal Care and Use Committee and approved by the President of the Nippon Medical School (approval number: 27-033).

### Drug treatments

OXT (Sigma-Aldrich, St Louis, MO, USA) and L-368,899 hydrochloride (Tocris Bioscience, Abingdon, UK), an OXT receptor antagonist, were dissolved in saline and subcutaneously injected into the mice. L-368,899 (10 mg/kg) was injected into pregnant WT female mice at GD19. The average length of time between injection and delivery was 6.9 h ± 26 min (*n* = 31). In the prenatal treatments, OXT (83 IU [0.2–0.26 mg]/kg) was injected into pregnant WT female mice 5 min before C/S (preOXT-C/S). In postnatal treatments, OXT (31.3 mIU/pup) was injected 30 min after delivery into naturally delivered or C/S-delivered pups as well as pups born to L-368,899-treated or OXT-KO dams (ND-OXT, C/S-OXT, LND-OXT, or IVF-OKO-OXT, respectively). The treatment doses of L-368,899 and OXT were determined based on previous reports^27^. Saline vehicle was injected into all mice 30 min after delivery for the non-treated animals (ND, C/S, LND, IVF-WT, or IVF-OKO).

### Behavioral tests

All behavioral tests were performed between 07:30 and 15:00 during postnatal weeks 8–11, except for the recording of USVs (performed at PD8).

The USVs of pups were measured at PD8. Pups weighing 4–6 g were examined. Each pup was separated from their foster ICR dam and placed in a plastic cup (diameter 5 cm; height 10 cm) in a sound-attenuated chamber. The USVs were recorded for 180 s with an UltraSoundGate 116Hb system (Avisoft Bioacoustics, Glienicke, Germany). The numbers of USV calls were counted using Avisoft-SASLab Pro (version 5.2) software (Avisoft Bioacoustics).

3-CSI test. The 3-CSI test was conducted as previously described^9^. Each subject mouse was acclimated to the test box by free exploration for 5 min before the test. Next, an unfamiliar younger WT C57BL/6J same-sex mouse (stranger mouse) that had no prior contact with the subject mouse was placed in a wire cage at one corner, while the other cage at the opposite corner remained empty. The subject mouse was placed in the middle chamber to freely move throughout all three chambers, and their behavior was recorded for 10 min. The time spent in the interaction zones (an area within 9 cm from the edge of each wire cage) was automatically measured as the approaching time using Image CSI software.

MB test. The MB test was conducted to detect repetitive and perseverative behavioral traits in mice^28^. Twenty blue glass marbles (diameter 15 mm) were arranged in a symmetrical 4 × 5 pattern on top of wood chip bedding (3 cm deep) in a standard mouse cage (27 cm × 44 cm × 19 cm). Each mouse was tested for 20 min, exploring under 110 lx illumination. The number of marbles that were buried (> two-thirds of the marble covered by bedding) was counted.

Rotarod test. The rotarod test was conducted to detect motor coordination and balance of mice. Mice weighing 25–30 g (males) or 19–23 g (females) were examined. Each mouse was placed on the rotarod with a rotating drum (3 cm diameter), which accelerated from 4 to 50 rpm over 300 s. The latency to fall off the rotarod was recorded for each trial. Six trials were performed at 50-min intervals.

All the apparatuses and analysis software, except for the USV and MB tests, were supplied by O’Hara & Co. Ltd. (Tokyo, Japan).

### Data analysis and producing figures

All data are expressed as the mean ± standard error of the mean (SEM). Data sets were first checked for normality using the Shapiro–Wilk test. Next, equal variances were checked using the *F* test in the case of two-group comparisons, or the Bartlett’s test in the case of multiple comparisons. If the data sets had normal distributions, parametric methods were used (two-way analysis of variance (ANOVA) test or Dunnett’s test). If not, non-parametric methods were used (Dunn’s test). To compare two groups, the two-tailed Student’s *t* test for data sets with equal variances (Figs. 1a, 3a,b,f, and Supplementary Figs. 1i,k, 2e, 4a,d,e) or Aspin–Welch *t* test for data sets with non-equal variances (Supplementary Figs. 1j, 4b,c) were used. For the 3-CSI tests, the two-tailed Wilcoxon matched-pairs signed-rank test was used, because the time spent with the stranger mouse and with the empty cage were paired data. Cohen’s *d* values were calculated to express the effect size for significant pairwise comparisons (Figs. 1b,c, 2d, 3c,d, and Supplementary Fig. 2d) as previously described^9^. To compare three or more groups, Dunnett’s test was used (Figs. 2a, 3e,g, and Supplementary Fig. 1e,f, 2a, 3b,c,g). Dunn’s test was used in Fig. 2c and Supplementary Fig. 1a–d,g,h, 2b,c,g,h, 3a,d–g. Furthermore, two-way ANOVA tests were used in Fig. 3g,h and in Supplementary Figs. 1f and 2f. All analyses were performed using GraphPad Prism software (GraphPad Software, San Diego, CA, USA). *P*-values < 0.05 were considered statistically significant. All statistical results are described in Supplemental Table 1. Further information can be found in the Supplemental information. All figures were produced using Adobe Illustrator software (Adobe Inc. San Jose, CA, USA).

## Supplementary Information

Supplementary Information.

## Data Availability

The datasets generated during and/or analyzed during the current study are available from the corresponding author on reasonable request.
