# Supplementary material for: Cesarean section delivery is a risk factor of autism-related behaviors in mice
Source: Sci Rep. 2021 Apr 26;11:8883. doi: 10.1038/s41598-021-88437-8 (PMC8076189; doi:10.1038/s41598-021-88437-8)
Supplement: Supplementary file 1 — Supplementary Information. [file 41598_2021_88437_MOESM1_ESM.pdf]

Cesarean section delivery is a risk factor for autism-related behaviors in mice

Masatoshi Nagano[1,*], Fumihito Saitow[1], Shinpei Higo[2], Makoto Uzuki[3], Yasunori Mikahara[1], Toshio Akimoto[3], Hitoshi Ozawa[2], Katsuhiko Nishimori[4], and Hidenori Suzuki[1]

1 Department of Pharmacology, Graduate School of Medicine, Nippon Medical School, 1-1-5 Sendagi, Bunkyo-ku, Tokyo, Japan

2 Department of Anatomy and Neurobiology, Graduate School of Medicine, Nippon Medical School

3 Division of Laboratory Animal Science, Nippon Medical School

4 Department of Obesity and Internal Inflammation, Fukushima Medical University, Hikarigaoka 1, Fukushima City, Fukushima, Japan

E-mails:

Masatoshi Nagano: nagano@nms.ac.jp

*Corresponding author: Masatoshi Nagano, PhD.

Tel: +81-3-3822-2131 ext. 5277; Fax: +81-3-5814-1684

Supplemental information

Figures and legends

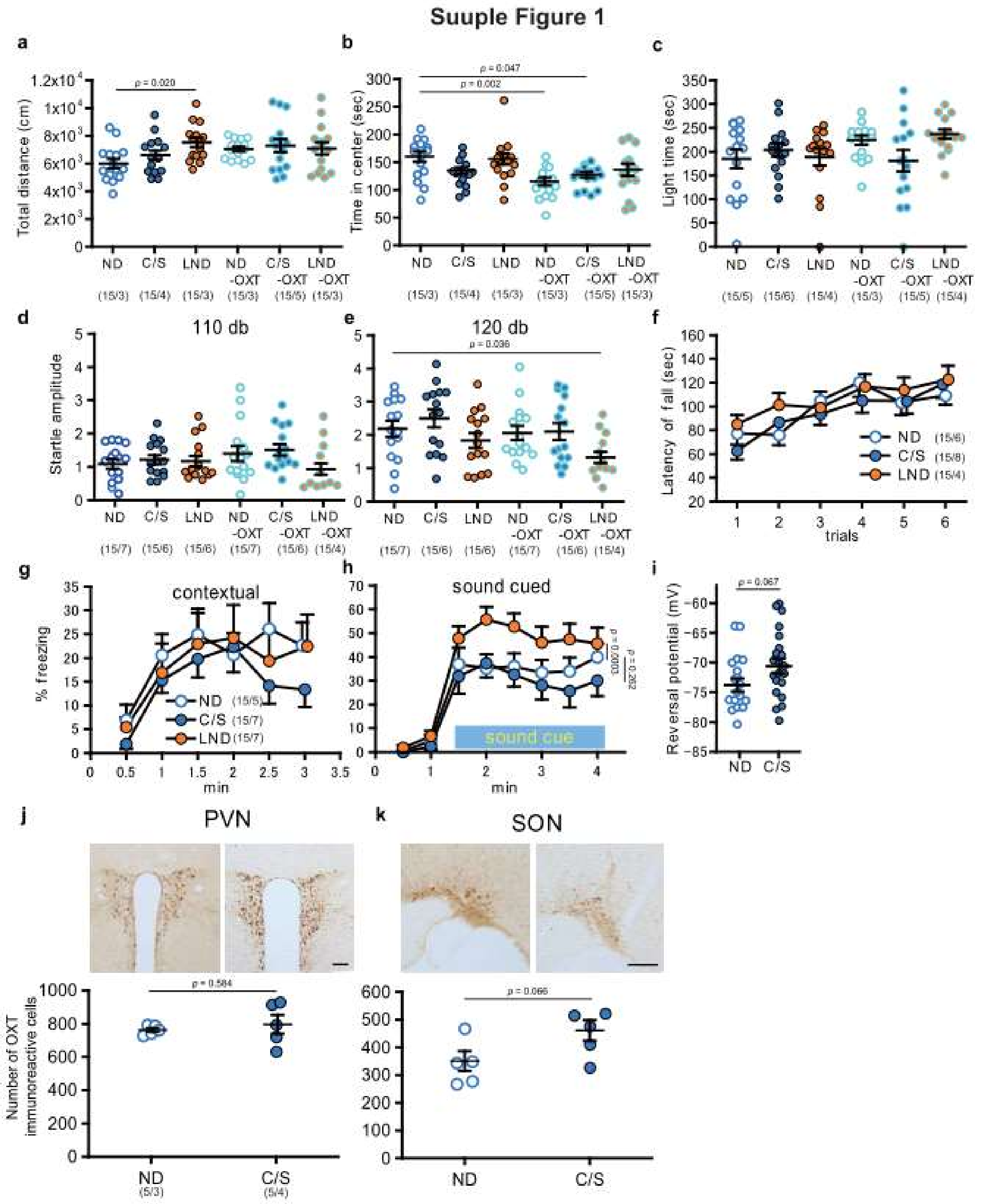

Supplemental Fig. 1. Effects of C/S delivery, prenatal OXT-R antagonist, and postnatal OXT on male offspring.

a Total distance in the OF test. **b** Time spent in the center area in the OF test. **c** Time spent in the light chamber of the L/D test. **d, e** Strength of the acoustic startle response (**d**: 110 dB, **e**: 120 dB). **f** Latency to fall from the rod in the rotarod test. **g, h** Percentage ratio of freezing in the acoustic FC test (**g**: contextual at day 2; **h**: sound cued at day 3). **i** Reversal potentials in hippocampal neurons of PD13–14 mice (ND –73.3 ± 1.1 mV vs. C/S –70.2 ± 1.2 mV). The number of examined hippocampal slices was 18 in the ND mice and 21 in the C/S mice (derived from three mice in different litters for each group). **j, k** Comparison of the numbers of OXT-immunoreactive cells in the hypothalamus (**j**: PVN, **k**: SON). Upper panels show representative photos. Scale bars in the photos indicate 100 μm in both the PVN and the SON. Data represent mean ± SEM. The numbers in parentheses indicate the number of mice tested and their litters (mice/litters) in each group.

Suuple Figure 2

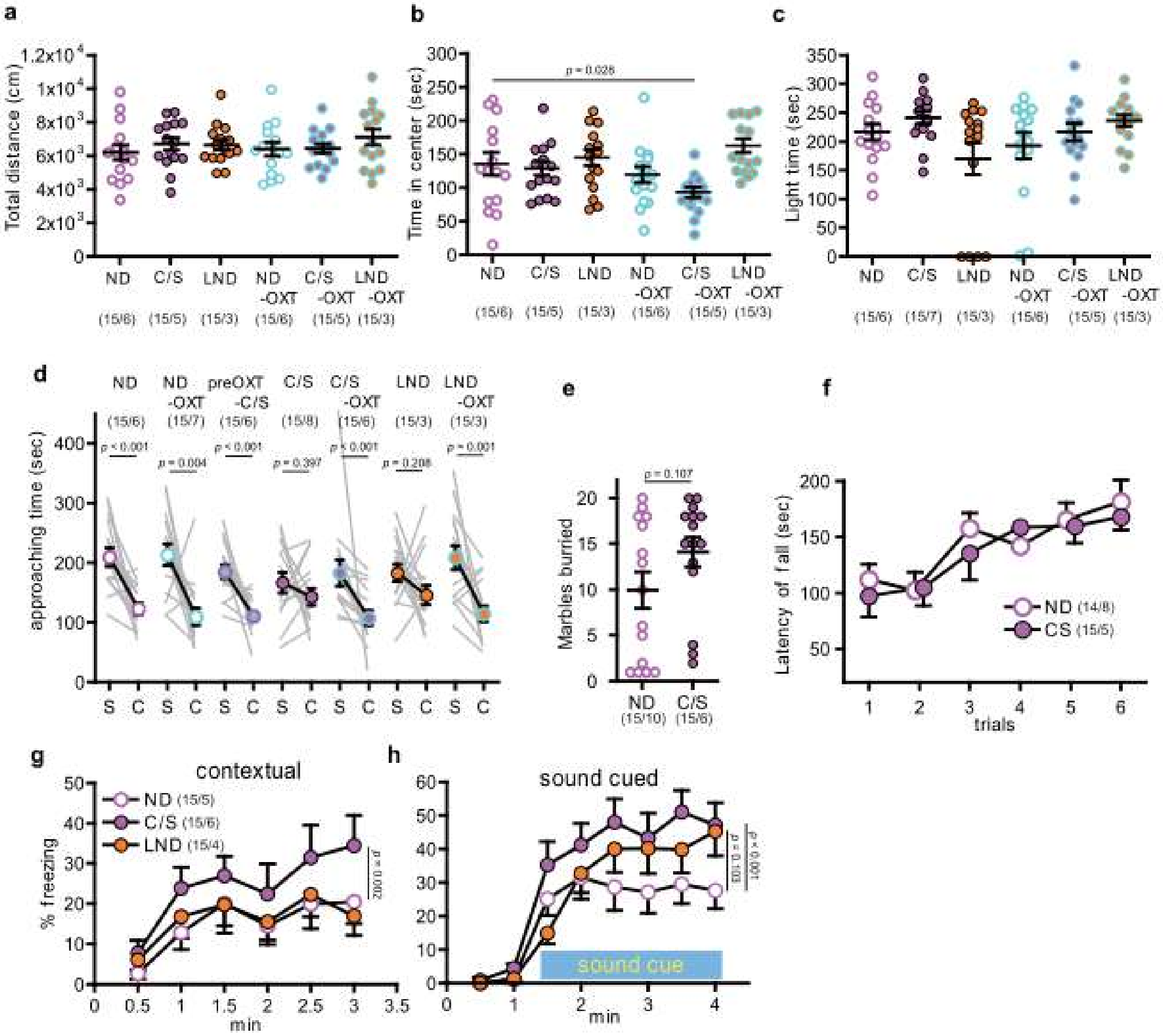

Supplementary Fig. 2. Effects of C/S delivery, prenatal OXT-R antagonist, and postnatal OXT on female offspring behaviors.

a Total distance in the OF test. **b** Time spent in the center area in the OF test. **c** Time spent in the light chamber of the L/D test. **d** Approaching time to the stranger cage (S) and the empty cage (C) in the 3-CSI test. **e** Number of marbles buried in the MB test. **f** Latency to fall from the rod in the rotarod test. **g, h** Percentage ratio of freezing in the

acoustic FC test (**g**: contextual at day 2, **h**: sound cued at day 3). Data represent mean ± SEM. The numbers in parentheses indicate the number of mice tested and their litters (mice/litters) in each group.

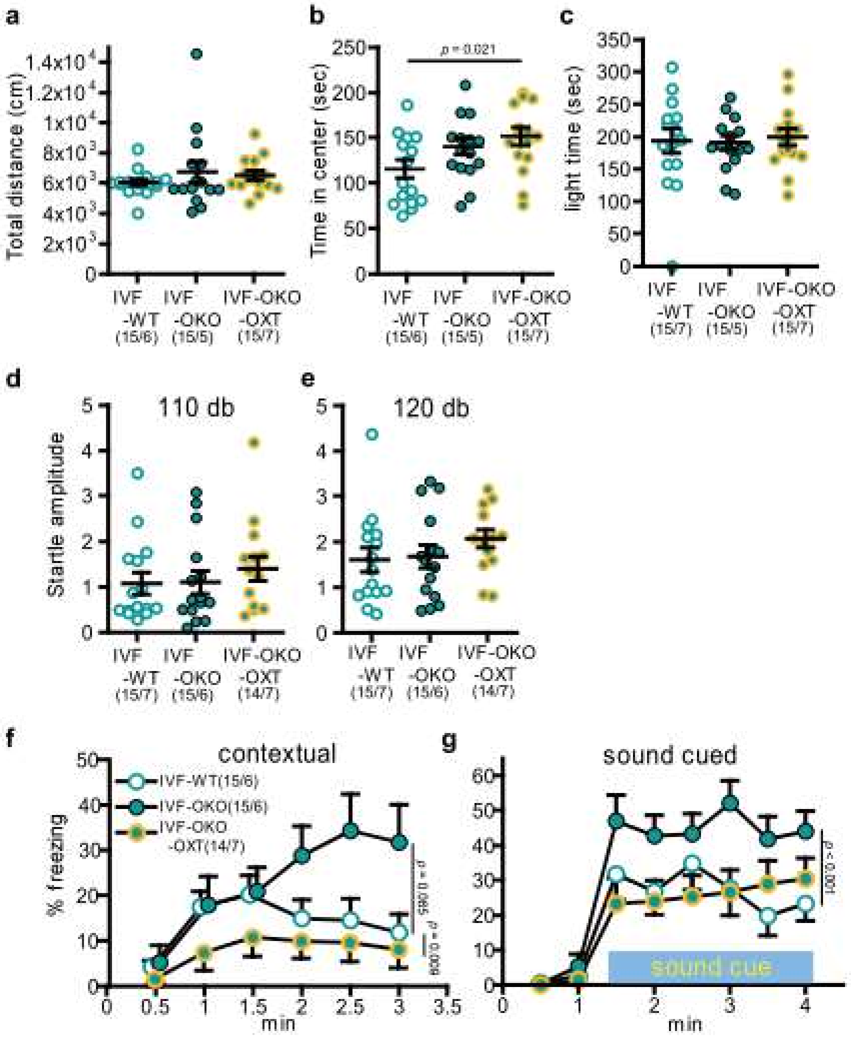

Supplementary Fig. 3. Effects of mothers lacking OXT on male offspring behaviors. **a** Total distance in the OF test. **b** Time spent in the center area in the OF test. **c** Time spent in the light chamber of the L/D test. **d, e** Strength of the acoustic startle response (**d**: 110 dB, **e**: 120 dB). **f, g** Percentage ratio of freezing in the acoustic FC test. (**f**: contextual at day 2, **g**: sound cued at day 3). Data represent mean ± SEM. The numbers in parentheses indicate the number of mice tested and their litters (mice/litters) in each group.

Suuple Figure 4

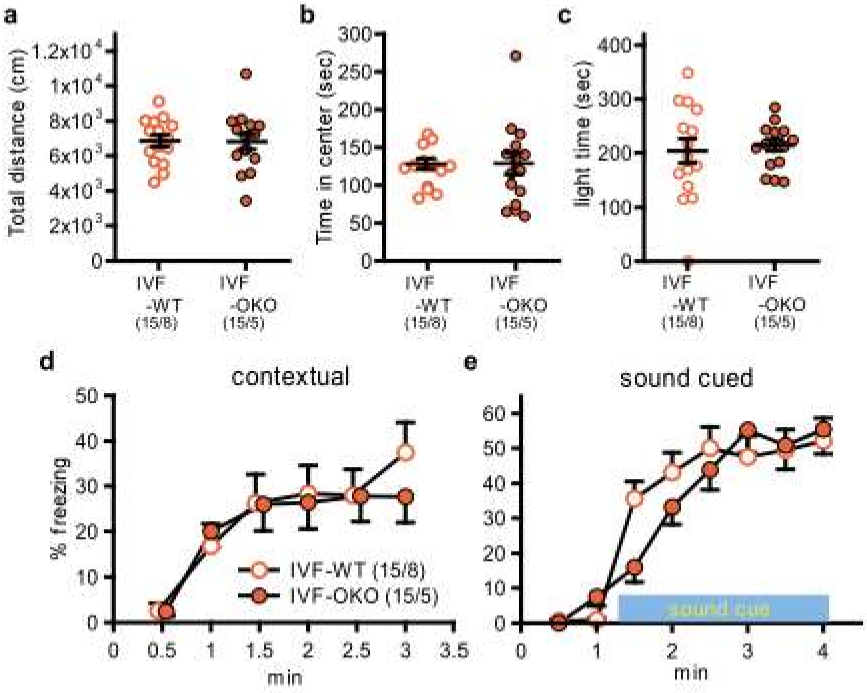

Supplementary Fig. 4. Effects of mothers lacking OXT on female offspring behaviors. **a** Total distance in the OF test. **b** Time spent in the center area in the OF test. **c** Time spent in the light chamber of the L/D test. **d, e** Percentage ratio of freezing in the acoustic FC test (**d**: contextual at day 2, **e**: sound cued at day 3). Data represent mean ± SEM. The numbers in parentheses indicate the number of mice tested and their litters (mice/litters) in each group.

Methods

Behavioral tests. Open field (OF) test. The OF test was performed before the other behavioral tests. The plastic open field chamber was 50 cm (length) × 50 cm (width) × 40 cm (height). The field was illuminated at 40 lux. Behaviors were recorded for 15 min using a CCD camera connected to a computer. The traveled distance and percentage of time spent in the center of the field were measured automatically using Image OF software. The center of the field was defined as a central square of 30 cm × 30 cm.

Light/dark transition (LD) test. The apparatus consisted of a cage (21 cm × 42 cm × 25 cm) divided into two sections of equal size by a partition with a door (4.5 cm × 3 cm). One chamber was brightly illuminated (600 lux), whereas the other chamber was dark (8 lux). Mice were placed in the dark chamber. After 5 s, the door was opened for 10 min and the mice were allowed to move freely between the two chambers. The time spent on each side and the latency of the first transition to the light chamber were recorded and analyzed automatically using Image LD software.

Acoustic startle responses. These responses were measured with 110 or 120 dB stimulus sounds of 50 ms duration. Mice were placed in a plastic cylinder and left undisturbed for 10 min for acclimation in a sound-attenuated chamber with a 70 dB background noise. Next, the startle responses were recorded. After receiving 60 stimulus sounds at 5-s intervals, the average amplitude of the startle responses was calculated at sounds of about 110 and 120 dB. The sound presentation and measurement of the responses was conducted using AnimalStartle software. This test was conducted with male mice only.

Acoustic contextual and cued fear-conditioning (FC) test. On the first day, each mouse was placed in a transparent chamber (26 cm × 34 cm × 29 cm) inside a sound-attenuated chamber with white walls and allowed to explore freely for 3 min. A sound of 70 dB

and 10 kHz, which served as the conditioned stimulus (CS), was presented for 20 s, followed by a mild (2 s, 0.3 mA) foot shock, which served as the unconditioned stimulus (US). One more CS–US pairing was presented with a 2-min interstimulus interval. On day 2, context testing was conducted in the same chamber for 6 min. On day 3, cued testing with altered context was conducted using a white opaque plastic chamber (26 cm × 34 cm × 29 cm) inside a sound-attenuated chamber with black walls for 6 min. The CS was presented during the last 5 min. Data acquisition, control of stimuli (i.e., tones and shocks), and data analysis were performed automatically using TimeFZ1 software. Images were captured at 2 frames/s. For each pair of successive frames, the area (pixels) by which the mice moved was measured. When this area was below 20 pixels, the behavior was judged as 'freezing'. When the amount of area equaled or exceeded the 20-pixel threshold, the behavior was considered as 'non-freezing'. 'Freezing' that lasted less than 2 s was not included in the analysis.
All of the apparatuses and analysis software were supplied by O'Hara & Co. Ltd. (Tokyo, Japan).

Electrophysiological analysis. Hippocampal slice preparation. Thin hippocampal slices were prepared from PD13–14 mice. Animals were deeply anesthetized with halothane inhalation (approximately 2% in air, v/v), and the brains were rapidly removed. Transverse slices (300 μm thick) were prepared from the mid-hippocampus using a tissue slicer (VT1200, Leica Microsystems, Wetzlar, Germany) at 4°C in Na^+-deficient saline that contained the following: 299.2 mM sucrose, 3.4 mM KCl, 0.3 mM $CaCl_2$, 3.0 mM $MgCl_2$, 10 mM HEPES, 0.6 mM NaH_2PO_4, and 10 mM glucose (Kusakari et al., PMID: 25713104). The slices were incubated at 30°C for 10 min and then maintained in a submerged chamber for more than 1.5 h in artificial cerebrospinal fluid (ACSF) that

contained the following: 125 mM NaCl, 2.5 mM KCl, 2.0 mM $CaCl_2$, 1.3 mM $MgCl_2$, 26.0 mM $NaHCO_3$, 1.25 mM NaH_2PO_4, and 11 mM glucose. Each slice was placed in a submersion-type recording chamber with ACSF flowing at 1.4 mL/min at 30°C. In the electrophysiological experiments, the slices were superfused with ACSF containing 500 nM tetrodotoxin and 2 μM CGP55845 to eliminate both Na^+-dependent synaptic transmissions and $GABA_B$ receptor activation.

Patch-clamp recordings. Hippocampal CA3 pyramidal neurons were visually identified under infrared differential interference contrast imaging using a water-immersion objective (40×, NA = 0.80; Olympus, Tokyo, Japan). Perforated patch-clamp recordings were performed as described previously[1]. In brief, patch pipettes had a resistance of 5.0–6.0 MΩ when filled with a high Cl^- internal solution that contained the following components (in mM): 150 KCl and 10 Na-HEPES (pH 7.35 with KOH), with 20 μM Alexa Fluor 594 to check whether the patch-membrane was ruptured. After getting a GΩ seal, the holding potential was set at –70 mV, so that before membrane perforation, the transpatch potential was close to 0 mV, and after perforation, the cell was roughly at resting potential. The perforation progress was monitored by evaluating the access resistance, deduced from the amplitude of the capacitive transients in response to repeated 10 mV hyperpolarizing steps every 30 s. The access resistance decreased and the apparent input capacitance increased to stabilize within a delay of 40 min after establishment of the seal. After reaching stable perforation, the reversal potential of $GABA_A$ receptor ($GABA_AR$)-mediated responses were determined by current–voltage (I-V) relationships before and during the exogenous application of the $GABA_AR$ agonist isoguvacine. First, 10 μM isoguvacine was focally applied using air pressure (3–6 psi) through a micropipette (1–2 μm diameter). The I-V relationships were obtained by

applying voltage ramps (–110 to –40 mV, 400 ms). Membrane currents were acquired and controlled using the Axon 700B Multiclamp Amplifier (Molecular Devices, San Jose, CA, USA) and pClamp10 acquisition software (Molecular Devices). Data were low-passed at 2 kHz using a Bessel filter and acquired at 20 kHz. Obtained data were analyzed using Clampfit (Molecular Devices) and Kyplot (Kyenslab, Tokyo, Japan) software.

Immunohistochemical analysis. Mice were transcardially perfused with 10 mL of phosphate-buffered saline (PBS, 0.1 M phosphate buffer [PB] containing 0.9% NaCl, pH 7.4) followed by 30 mL of 4% paraformaldehyde in 0.1 M PB (pH 7.4), under deep anesthesia with a mixture of medetomidine, midazolam, and butorphanol (0.3 mg/kg, 4 mg/kg and 5 mg/kg, respectively, i.p.). Brains were post-fixed in the same fixative at 4°C for 16 h, and then transferred into 0.1 M PB containing 20% sucrose for cryoprotection. Three series of serial coronal sections were cut at 25 µm thickness using a cryostat (Leica 3050, Heidelberg, Germany) and collected in PBS.

Immunohistochemistry for oxytocin was performed using a streptavidin–horseradish peroxidase-based staining method using a Histofine SAB-PO Kit (Nichirei Corporation, Tokyo, Japan) with 3,3'-diaminobenzidine (DAB) as a chromogen. A series of free-floating brain sections from each animal were treated with 0.03% H_2O_2 in PBS, followed by blocking in 5% normal rabbit serum. Sections were incubated at 4°C for 24 h with a primary anti-oxytocin antibody at a dilution of 1:10,000 (AB911; Merck Millipore, Billerica, MA, USA) in PBS containing 0.3% (v/v) Triton X-100 (PBST). Oxytocin neurons in the PVN and SON were visualized following the protocol supplied by the manufacturer of the Histofine kit. After color development in the chromogen solution (0.05 M Tris buffer containing DAB), sections were mounted on MAS-coated

slides (Matsunami Glass Ind., Osaka, Japan) and cover-slipped with Permount (Fisher Scientific Inc, Waltham, MA, USA). Images of each section were acquired using a BX-51 microscope (Olympus). Oxytocin-positive neurons in each section were counted using ImageJ with the Cell Counter plugin. The analyzer was blinded to the experimental groups.

References

1. Satoh, H., Qu, L,. Suzuki, H., Saitoh, F. Depolarization-induced depression of inhibitory transmission in cerebellar Purkinje cells. *Physiol. Rep.* **1**, e00061. (2013)

Supplemental Table 1. Statistical results

Figure	Group	sample number (mice/litters)	normal distribution(No: at least more than one group)	Shapiro-Wilk test	F test, Bartlett test n.a.: not applicable	ANOVA	Statistics		*p* value	Cohen's *d*
Fig. 1a	ND	38/7 (male:19; female19)	No	0.71	F test $p = 0.636$		two-tailed Student's *t*- test		0.039	
	C/S	38/9 (male:19; female19)		0.02						
Fig. 1b	ND	15/5	Yes	S: 0.10; C: 0.59	n.a.		two-tailed Wilcoxon matched-pairs signed rank test (S vs. C)		0.0002	2.06
	C/S	15/4		S: 0.10; C: 0.10					0.751	0.16
Fig. 1c	ND	15/6	No	S: 0.04; C: 0.94	n.a.		two-tailed Wilcoxon matched-pairs signed rank test (S vs. C)		0.030	0.97
	C/S	15/6		S: 0.75; C: 0.04					0.303	0.54
Fig. 2a	ND	40/10 (male:20; female20)	Yes	0.69	Bartlett test $p = 0.435$		Dunnett's test	-	-	
	ND-OXT	40/12 (male:20; female20)		0.17				ND vs.ND-OXT	0.998	
	pre-OXT-C/S	40/9 (male:20; female20)		0.59				ND vs. pre-OXT-C/S	0.263	
	C/S	40/11 (male:20; female20)		0.93				ND vs. C/S	0.016	
	C/S-OXT	40/10 (male:20; female20)		0.38				ND vs. C/S-OXT	0.805	
	LND	40/11 (male:20; female20)		0.48				ND vs. LND	0.009	
	LND-OXT	40/11 (male:20; female20)		0.15				ND vs. LND-OXT	0.696	
Fig. 2b	ND	15/4	Yes	S: 0.17; C: 0.98	n.a.		two-tailed Wilcoxon matched-pairs signed rank test (S vs. C)		0.001	1.78
	ND-OXT	15/4	Yes	S: 0.86; C: 0.63					0.0003	2.05
	pre-OXT-C/S	15/4	Yes	S: 0.45; C: 0.43					0.0006	1.75
	C/S	15/6	Yes	S: 0.70; C: 0.45					0.268	0.43
	C/S-OXT	15/5	Yes	S: 0.30; C: 0.50					< 0.0001	2.28
	LND	15/6	No	S: 0.69; C: 0.05					0.804	0.30
	LND-OXT	15/4	Yes	S: 0.11; C: 0.15					0.0006	2.34
Fig. 2c	ND	15/9	No	0.048	Bartlett test $p = 0.012$		Dunn's test	-	-	
	ND-OXT	15/6		0.47				ND vs.ND-OXT	> 0.9999	
	pre-OXT-C/S	15/6		0.047				ND vs. pre-OXT-C/S	> 0.9999	
	C/S	15/4		0.02				ND vs. C/S	0.0006	
	C/S-OXT	15/5		0.07				ND vs. C/S-OXT	> 0.9999	
	LND	15/7		0.07				ND vs. LND	0.035	
	LND-OXT	15/7		0.28				ND vs. LND-OXT	> 0.9999	
Fig. 3a	IVF-WT	15/8	Yes	0.21	F test $p = 0.815$		two-tailed Student's *t-test*		0.681	
	IVF-OKO	15/6		0.75						
Fig. 3b	IVF-WT	15/8	Yes	0.10	F test $p = 0.79$		two-tailed Student's *t-test*		0.006	
	IVF-OKO	15/8		0.44						
Fig. 3c	IVF-WT	15/7	Yes	S: 0.39; C: 0.31	n.a.		two-tailed Wilcoxon matched-pairs signed rank test (S vs. C)		< 0.0001	3.70
	IVF-OKO	15/5	No	S: 0.59; C: 0.004					0.124	0.40
	IVF-OKO-OXT	15/7	No	S: 0.37; C: 0.04					0.0009	2.21
Fig. 3d	IVF-WT	15/8	No	S: 0.01; C: 0.46	n.a.		two-tailed Wilcoxon matched-pairs signed rank test (S vs. C)		0.0006	1.57
	IVF-OKO	15/5	Yes	S: 0.39; C: 0.31					0.0003	1.64
Fig. 3e	IVF-WT	15/9	Yes	0.74	Bartlett test $p = 0.053$		Dunnett's test	-	-	
	IVF-OKO	15/5		0.19				IVF-WT vs. IVF-OKO	0.001	
	IVF-OKO-OXT	15/8		0.48				IVF-WT vs. IVF-OKO-OXT	0.492	
Fig. 3f	IVF-WT	12/5	Yes	0.07	F test $p = 0.913$		two-tailed Student's *t-test*		0.223	
	IVF-OKO	15/8		0.36						
Fig. 3g	IVF-WT	15/8	supposed		n.a.	interaction: $F_{10,205} = 1.037$, $p = 0.413$	Dunnet's test	-	-	
	IVF-OKO	15/5				trial: $F_{5,205} = 13.83$, $p < 0.0001$		IVF-WT vs. IVF-OKO	0.003	
	IVF-OKO-OXT	14/8				group: $F_{2,41} = 5.913$, $p = 0.006$		IVF-WT vs. IVF-OKO-OXT	0.044	
Fig. 3h	IVF-WT	12/5	supposed		n.a.	interaction: $F_{5,120} = 1.709$, $p = 0.138$				
	IVF-OKO	14/7				trial: $F_{5,120} = 9.088$, $p < 0.0001$<br>group: $F_{1,24} = 0.399$, $p = 0.534$				

Figure	Group	sample number (mice/litters)	normal distribution (No: at least more than one group)	Shapiro-Wilk test	F test, Bartlett test n.a.: not applicable	ANOVA	Statistics		*p* value
Fig. S1a	ND	15/3	Yes	0.59	Bartlett test *p* = 0.025		Dunnett's test	-	-
	C/S	15/4		0.41				ND vs. C/S	0.679
	LND	15/3		0.69				ND vs. LND	0.019
	ND-OXT	15/3		0.26				ND vs.ND-OXT	0.179
	C/S-OXT	15/5		0.15				ND vs. C/S-OXT	0.059
	LND-OXT	15/3		0.25				ND vs. LND-OXT	0.150
Fig. S1b	ND	15/3	No	0.50	Bartlett test *p* = 0.05		Dunn's test	-	-
	C/S	15/4		0.94				ND vs. C/S	0.230
	LND	15/3		0.04				ND vs. LND	> 0.999
	ND-OXT	15/3		0.997				ND vs.ND-OXT	0.002
	C/S-OXT	15/5		0.19				ND vs. C/S-OXT	0.047
	LND-OXT	15/3		0.43				ND vs. LND-OXT	0.415
Fig. S1c	ND	15/5	No	0.74	Bartlett test *p* = 0.008		Dunn's test	-	-
	C/S	15/6		0.95				ND vs. C/S	> 0.999
	LND	15/4		0.004				ND vs. LND	> 0.999
	ND-OXT	15/3		0.997				ND vs.ND-OXT	0.854
	C/S-OXT	15/5		0.21				ND vs. C/S-OXT	> 0.999
	LND-OXT	15/4		0.73				ND vs. LND-OXT	0.289
Fig. S1d	ND	15/7	No	0.26	Bartlett test *p* = 0.352		Dunn's test	-	-
	C/S	15/6		0.42				ND vs. C/S	> 0.999
	LND	15/6		0.008				ND vs. LND	> 0.999
	ND-OXT	15/7		0.08				ND vs.ND-OXT	> 0.999
	C/S-OXT	15/6		0.19				ND vs. C/S-OXT	0.579
	LND-OXT	15/4		0.01				ND vs. LND-OXT	> 0.999
Fig. S1e	ND	15/7	Yes	0.52	Bartlett test *p* = 0.650		Dunnett's test	-	-
	C/S	15/6		0.43				ND vs. C/S	0.821
	LND	15/6		0.33				ND vs. LND	0.713
	ND-OXT	15/7		0.20				ND vs.ND-OXT	0.993
	C/S-OXT	15/6		0.07				ND vs. C/S-OXT	0.999
	LND-OXT	15/4		0.27				ND vs. LND-OXT	0.036
Fig. S1f	ND	15/6	supposed		n.a.	interaction: $F_{10, 210} = 0.362$, p =0.904	Dunnett's test	-	-
	C/S	15/8				trial: $F_{5, 210} = 12.93$, p <0.0001		ND vs. C/S	0.930
	LND	15/4				group: $F_{2, 42} = 0.578$, p =0.565		ND vs. LND	0.687
Fig. S1g	ND	15/5	No	0.0005 (1 - 3 min)	Bartlett test *p* = 0.0001		Dunn's test	-	-
	C/S	15/7		< 0.0001 (1 - 3 min)				ND vs. C/S	0.150
	LND	15/7		< 0.0001 (1 - 3 min)				ND vs. LND	0.179
Fig. S1h	ND	15/5	No	0.001 (1.5 - 4min)	Bartlett test *p* = 0.724		Dunn's test	-	-
	C/S	15/7		0.0002 (1.5 - 4min)				ND vs. C/S	0.229
	LND	15/7		0.08 (1.5 - 4min)				ND vs. LND	0.0007
Fig. S1i	ND	18 slices (3mice/3litters)	Yes	0.19	F test *p* = 0.421		two-tailed Student's *t-test*		0.067
	C/S	21 slices (3mice/3litters)		0.61					
Fig. S1j	ND	5/3	No	0.61	F test *p* = 0.013		two-tailed Welch-Aspin's *t-test*		0.584
	C/S	5/4		0.59					
Fig. S1k	ND	5/3	Yes	0.32	F test *p* = 0.956		two-tailed Student's *t-test*		0.066
	C/S	5/4		0.38					

Figure	Group	sample number (mice/litters)	normal distribution(No: at least more than one group)	Shapiro-Wilk test	F test, Bartlett test n.a.: not applicable	ANOVA	Statistics		*p* value	Cohen's *d*
Fig. S2a	ND	15/6	Yes	0.70	Bartlett test p = 0.350		Dunnett's test	-	-	
	C/S	15/5		0.51				ND vs. C/S	0.726	
	LND	15/3		0.33				ND vs. LND	0.677	
	ND-OXT	15/6		0.51				ND vs.ND-OXT	0.368	
	C/S-OXT	15/5		0.94				ND vs. C/S-OXT	0.435	
	LND-OXT	15/3		0.63				ND vs. LND-OXT	0.997	
Fig. S2b	ND	15/6	No	0.65	Bartlett test p = 0.089		Dunn's test	-	-	
	C/S	15/5		0.26				ND vs. C/S	> 0.999	
	LND	15/3		0.66				ND vs. LND	> 0.999	
	ND-OXT	15/6		0.39				ND vs.ND-OXT	> 0.999	
	C/S-OXT	15/5		0.98				ND vs. C/S-OXT	0.079	
	LND-OXT	15/3		0.03				ND vs. LND-OXT	0.547	
Fig. S2c	ND	15/6	No	0.995	Bartlett test p = 0.0006		Dunn's test	-	-	
	C/S	15/7		0.86				ND vs. C/S	0.740	
	LND	15/6		0.0007				ND vs. LND	> 0.999	
	ND-OXT	15/6		0.0048				ND vs.ND-OXT	> 0.999	
	C/S-OXT	15/5		0.98				ND vs. C/S-OXT	> 0.999	
	LND-OXT	15/3		0.94				ND vs. LND-OXT	> 0.999	
Fig. S2d	ND	15/6	Yes	S: 0.74; C: 0.22	n.a.		two-tailed Wilcoxon matched-pairs signed rank test (S vs. C)		0.0006	1.34
	ND-OXT	15/7	No	S: 0.32; C: 0.02					0.004	1.66
	pre-OXT-C/S	15/6	Yes	S: 0.91; C: 0.73					0.0003	1.93
	C/S	15/8	Yes	S: 0.46; C: 0.26					0.397	0.41
	C/S-OXT	15/6	No	S: 0.0006; C: 0.80					< 0.0001	1.12
	LND	15/3	Yes	S: 0.98; C: 0.14					0.208	0.62
	LND-OXT	15/3	Yes	S: 0.63; C: 0.23					0.001	1.44
Fig. S2e	ND	15/10	No	0.02	F test p = 0.430		two-tailed Student's *t-test*		0.107	
	C/S	15/6		0.01						
Fig. S2f	ND	14/8	supposed		n.a.	interaction: $F_{5, 135}$ = 0.722, p =0.608<br>group: $F_{1, 27}$ = 0.180, p =0.675				
	C/S	15/5								
Fig. S2g	ND	15/5	No	< 0.0001 (1 - 3 min)	Bartlett test p = 0.005		Dunn's test	-	-	
	C/S	15/6		< 0.0001 (1 - 3 min)				ND vs. C/S	0.002	
	LND	15/4		< 0.0001 (1 - 3 min)				ND vs. LND	> 0.999	
Fig. S2h	ND	15/5	No	< 0.0001 (1.5 - 4 min)	Bartlett test p = 0.270		Dunn's test	-	-	
	C/S	15/6		0.02 (1.5 - 4 min)				ND vs. C/S	< 0.0001	
	LND	15/4		0.0002 (1.5 - 4 min)				ND vs. LND	0.150	

Figure	Group	sample number (mice/litters)	normal distribution(No: at least more than one group)	Shapiro-Wilk test	F test, Bartlett test	Statistics		*p* value
Fig. S3a	IVF-WT	15/6	No	0.06	Bartlett test $p = 0.0001$	Dunn's test	-	-
	IVF-OKO	15/5		0.002			IVF-WT vs. IVF-OKO	> 0.999
	IVF-OKO-OXT	15/7		0.61			IVF-WT vs. IVF-OKO-OXT	0.647
Fig. S3b	IVF-WT	15/6	Yes	0.15	Bartlett test $p = 0.935$	Dunnett's test	-	-
	IVF-OKO	15/5		0.88			IVF-WT vs. IVF-OKO	0.133
	IVF-OKO-OXT	15/7		0.43			IVF-WT vs. IVF-OKO-OXT	0.021
Fig. S3c	IVF-WT	15/7	Yes	0.33	Bartlett test $p = 0.086$	Dunnett's test	-	-
	IVF-OKO	15/5		0.81			IVF-WT vs. IVF-OKO	0.984
	IVF-OKO-OXT	15/7		0.88			IVF-WT vs. IVF-OKO-OXT	0.932
Fig. S3d	IVF-WT	15/7	No	0.004	Bartlett test $p = 0.903$	Dunn's test	-	-
	IVF-OKO	15/5		0.01			IVF-WT vs. IVF-OKO	> 0.999
	IVF-OKO-OXT	15/7		0.09			IVF-WT vs. IVF-OKO-OXT	0.293
Fig. S3e	IVF-WT	15/7	No	0.04	Bartlett test $p = 0.409$	Dunn's test	-	-
	IVF-OKO	15/5		0.12			IVF-WT vs. IVF-OKO	> 0.999
	IVF-OKO-OXT	15/7		0.43			IVF-WT vs. IVF-OKO-OXT	0.150
Fig. S3f	IVF-WT	15/6	No	< 0.0001 (1 - 3 min)	Bartlett test $p < 0.0001$	Dunn's test	-	-
	IVF-OKO	15/6		< 0.0001 (1 - 3 min)			IVF-WT vs. IVF-OKO	0.065
	IVF-OKO-OXT	14/7		< 0.0001 (1 - 3 min)			IVF-WT vs. IVF-OKO-OXT	0.009
Fig. S3g	IVF-WT	15/6	No	< 0.0001 (1.5 - 4 min)	Bartlett test $p = 0.922$	Dunn's test	-	-
	IVF-OKO	15/6		0.02 (1.5 - 4 min)			IVF-WT vs. IVF-OKO	< 0.0001
	IVF-OKO-OXT	14/7		< 0.0001 (1.5 - 4 min)			IVF-WT vs. IVF-OKO-OXT	0.810
Fig. S4a	IVF-WT	15/8	Yes	0.92	F test $p = 0.325$	two-tailed Student's *t-test*		0.923
	IVF-OKO	15/5		0.71				
Fig. S4b	IVF-WT	15/8	Yes	0.37	F test $p = 0.006$	two-tailed Welch-Aspin's *t-test*		0.951
	IVF-OKO	15/5		0.10				
Fig. S4c	IVF-WT	15/8	Yes	0.90	F test $p = 0.007$	two-tailed Welch-Aspin's *t-test*		0.571
	IVF-OKO	15/5		0.53				
Fig. S4d	IVF-WT	15/8	No	< 0.0001 (1 - 3 min)	F test $p = 0.210$	two-tailed Student's *t-test*		0.632
	IVF-OKO	15/5		0.0006 (1 - 3 min)				
Fig. S4e	IVF-WT	15/8	No	0.15 (1.5 - 4 min)	F test $p = 0.164$	two-tailed Student's *t-test*		0.464
	IVF-OKO	15/5		0.03 (1.5 - 4 min)				
